# COVID-19 in Italy: Comparison of CT Findings from Time Zero to the Delta Variant

**DOI:** 10.3390/microorganisms10040796

**Published:** 2022-04-09

**Authors:** Nicola Maggialetti, Ilaria Villanova, Annalisa Castrì, Chiara Noemi Greco, Francesco Inchingolo, Daniele Virgilio, Marco Moschetta, Angela Sardaro, Amato Antonio Stabile Ianora, Arnaldo Scardapane

**Affiliations:** 1 Department of Medical Science, Neuroscience and Sensory Organs (DSMBNOS), University of Bari “Aldo Moro”, 70124 Bari, Italy; n.maggialetti@gmail.com; 2 Interdisciplinary Department of Medicine, Section of Radiology and Radiation Oncology, University of Bari “Aldo Moro”, 70124 Bari, Italy; ila.vill@libero.it (I.V.); annalisacastri1990@gmail.com (A.C.); drdanielevirgilio@gmail.com (D.V.); marco.moschetta@uniba.it (M.M.); angela.sardaro@uniba.it (A.S.); amatoantonio.stabile@uniba.it (A.A.S.I.); arnaldo.scardapane@gmail.com (A.S.); 3 Interdisciplinary Department of Medicine, Section of Dental Medicine, University of Bari “Aldo Moro”, 70124 Bari, Italy; francesco.inchingolo@uniba.it; 4 Breast Unit, Policlinic Hospital of Bari, 70124 Bari, Italy

**Keywords:** COVID-19 disease, SARS-CoV-2, imaging, chest computed tomography (CT), COVID patterns, COVID variants, GGOs (ground-glass opacities), pneumonia

## Abstract

On 12 March 2020, the World Health Organization (WHO) declared the novel Coronavirus (CoV) disease a global Pandemic and an emerging risk. In order to understand patterns that are typical in COVID-19 pneumonia and track the evolution of the disease, the role of the chest computed tomography (CT) is pivotal. The impact of the illness as well as the efficiency of the therapy are also monitored carefully when performing this imaging exam. Coronaviruses, specifically CoV-2, as RNA viruses, have a tendency to frequently change their genome, giving the virus beneficial characteristics such as greater transmissibility, pathogenicity and the possibility to escape the previously acquired immunity. Therefore, genome evaluation became an extremely important routine practice worldwide. In particular, in Italy, four variants have been recognised and each of them represent a specific temporal wave of the disease. Hence, our goal was to describe imaging findings of COVID-19 pneumonia, specifically its most typical imaging identified during the period of our study, and to assess whether or not SARS-CoV-2 variants determine different CT patterns. Our analyses revealed that the SARS-CoV-2 genotype seems not to interfere with the severity of CT patterns and, in particular, bilateral Ground Glass Opacities (GGOs) are the most frequent findings in all COVID-19 waves.

## 1. Introduction

COVID-19 is a growing global health threat that causes important respiratory symptoms and has led to more than 5 million deaths around the World. It has rapidly spread worldwide, and thus, the WHO declared COVID-19 to be pandemic at the beginning of 2020 [1]. The structure of CoV is characterized by an enveloped positive single-stranded RNA [2]. The genetic sequence of SARS-CoV-2 was described as a beta-coronavirus that shares part of its genetics with SARS-CoV [3], and they both primarily infect the airways. Because of its long incubation period, which is known to be from 2 to 14 days and influenced by age group as well as the presence of comorbidities, its identification and tracing became a common challenge. In addition, the possibility of being infected with mild symptoms or without any, as in asymptomatic infections, increases the difficulty of controlling the spread of the virus [4,5].

Fever, cough, anosmia and dyspnea are described as being the most common symptoms of COVID-19. Additionally, asymptomatic infections and other symptoms, which are common to other viral respiratory diseases, have been reported. In the worst-case scenario, infection can result initially in interstitial pneumonia and then advance to severe acute respiratory syndrome (ARDS) and multiple organ dysfunction (MOF) until death [6,7].

Diagnosis is confirmed by nasopharyngeal or oropharyngeal swab tests based on highly specific positive reverse-transcription polymerase chain reaction (RT-PCR). Moreover, in order to support the diagnosis, chest CT and radiography are also regularly used to quantify severity and to assess the efficacy of treatment and its response. In addition, the potential role of pulmonary ultrasound in the diagnosis and in the long-term follow-up of COVID-19 is being evaluated in some medical centres [8,9].

Actually, the use of high-resolution unenhanced chest CT (HRCT) in COVID-19 patients is supported by current guidelines thanks to its high accuracy in detecting pulmonary changes of the viral pneumonia and also in allowing differential diagnoses [10]. In addition, today’s new techniques such as machine learning methods offer a great help in terms of the fast and accurate recognition of COVID-19 disease in both radiographs and CT images [11].

To ease the diagnosis, at present, technological tools such as an algorithm based on symptoms, cough sounds and hematological parameters, as well as a cloud-based smartphone application platform, are now being developed to combat the spread of the virus [12,13].

There is no resolution therapy available, but current treatments are based on supporting therapy in order to prevent intensive care unit hospitalization [14]. Therefore, the beginning of a vaccination campaign seems to be the safest and most effective modality to prevent COVID-19 illness and death, and the best way to deal with possible virus-related genotype variants [15].

Chest CT images of COVID-19 patients typically present as multifocal patchy ground-glass opacities (GGOs) with interlobular septal and vascular thickening together with consolidation. Those are most likely to be located in the lower area of the lung parenchyma. Incidence rates of GGOs and consolidation are about 86% and 29%, respectively [16]. Opacities are usually described as patchy, rounded, triangular or linear. Another typical sign is the triangular or angular GGOs under the pleura, associated with the thickening of the internal interlobular septa [17]. Even if rare, it is also possible to find lung cavitation, multiple nodules, pleural effusions, tree-in-bud opacities, honeycombing, traction bronchiectasis and lymphadenopathy [16]. Known patterns can also be observed e.g., “crazy paving” (a diffuse ground-glass attenuation with the superimposition of interlobular septal thickening and intralobular lines) [18] or the “reverse halo sign” (a focal, rounded area of ground-glass surrounded by a ring of consolidation) [19].

Coronaviruses, specifically CoV-2, as with other RNA viruses, constantly change their genome so that the virus can acquire selective advantages such as greater transmissibility, greater pathogenicity with more severe forms of disease and the possibility to escape previously acquired immunity. Consequently, these mutations became a cause for concern and must be monitored carefully. In particular, four variants have been recognised in Italy and each one is predominant in a specific wave of the disease [20]. Virus variants have different impacts on the contagiousness, clinical presentations, severity of symptoms and outcomes of the disease.

In this context, CT has become a rapid and available tool in the emergency room (ER) in contrast with the genomic sequencing of SARS-CoV-2, which is less accessible and not always manageable. Starting from this assumption the aim of the study was to demonstrate whether CoV-2 variants determine variable findings in CT imaging exams in order to facilitate the differential diagnoses among them.

## 2. Materials and Methods

### 2.1. Study Sample

In this cohort study, we evaluated a population of 461 patients (mean age: 65 years; 285 male, 176 female; median age range: from 18 to 104 years) with COVID-19 pneumonia who underwent a first admission chest CT scan at the “Policlinico di Bari” COVID Hospital, Italy, from March 2020 to October 2021. The local ethical committee approved the study.

**Figure 1:**
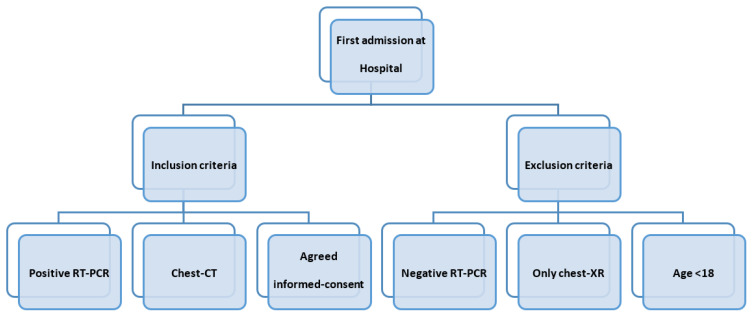
Flow chart: inclusion and exclusion criteria.

The inclusion criteria were: (a) patients tested positive to real-time reverse transcriptase polymerase chain reaction (RT-PCR) for SARS-CoV-2 of nasopharyngeal swabs, (b) chest-CT was performed at first hospital admission; (c) participants gave informed-consent for chest-CT. We evaluated the patients who showed up in the ER with respiratory symptoms regardless of comorbidities, due to the difficulty of obtaining their complete histories with their urgent conditions. The exclusion criteria were: (a) patients tested negative to real-time reverse transcriptase polymerase chain reaction (RT-PCR) for SARS-CoV2 of nasopharyngeal swabs, (b) patients had only a chest-x ray at first admission at the hospital (c) age < 18 years old because they were addressed to the paediatric hospital (Figure 1).

Confirmation of the variant strain was accomplished by means of genomic sequencing of SARS-CoV-2 from positive random samples from hospitalized patients performed by the department of Tropical and Infectious Disease at Policlinico of Bari.

### 2.2. Chest and Scanning Protocol

With the purpose of studying the lung parenchyma, non-contrast CT examinations were performed using the Siemens Somatom Definition DS CT scanner with the following acquisition parameters: slice thickness 0.75 mm, tube voltage 100 kVp, 38 mAs, rotation time 0.33 s, pitch 1.1. When possible, considering patient conditions, breath-holding at full inspiration was required for the acquisition of images. Reconstruction of images was performed with a slice thickness of 1 mm (to optimize the signal-to-noise-ratio) in mediastinal and parenchymal windows.

Faster exams with standard chest CT protocols were preferred due to the critical conditions of patients.

### 2.3. Imaging Assessment

The acquired CT data were collected through our institutional PACS system (Carestream Health, Rochester, NY, USA). Images were then analysed using Multiplanar Reformatting (MPR) and 3D Maximum Intensity Projection (3D MIP) and displayed with two grey-scale windows, namely the lung window and mediastinal window settings. Visual assessments were performed independently by 2 radiologists (A.S. and N.M., with 23 and 12 years of experience, respectively); disagreements were resolved by open discussion and consensus from all the authors.

The findings were described according to the Fleischner Society glossary and in accordance with the article from Morelli, C., ‘The Multifaceted COVID-19: CT Aspects of Its Atypical Pulmonary and Abdominal Manifestations and Complications in Adults and Children. A Pictorial Review’ [21].

In particular, we considered: (a) GGOs (GROUND GLASS OPACITIES), (b) CONSOLIDATION, (c) SUBPLEURAL BANDS and (d) PLEURAL EFFUSION.

A semi-quantitative CT-score system was used to estimate the involvement of lung lesions. This score ranged from 0 to 5 points for each lobe, reaching a maximum of 25 points for the sum of both lungs. Each point from 0 to 5 represented the percentage volume of lung lobe implication: 1 point—<5% involvement; 2 points—5–25% involvement; 3 points—26–49% involvement; 4 points—50–75% involvement; 5 points—>75% involvement [22]. Low score levels were considered when the sum was inferior to 15 points; the severity increased from 15 to the highest score of 25 points.

### 2.4. Statistical Analysis

All statistical analyses were performed using IBM SPSS Statistics Software (version 26; IBM, Armonk, NY, USA).

The continuous variables are expressed as mean values ± standard deviation (SD), and the categorical variables are given as percentages.

One-way ANOVA (Analysis of Variance) tests were performed to compare the effects of age and CT semi-quantitative scores between the four waves considered. A forward stepwise logistic regression analysis was used to assess the previously cited univariate predictors independently associated with population age and CT scores. If the *p*-value was <0.005, the results were considered to be statistically significant, meaning that the test hypothesis was false or needed to be rejected.

## 3. Results

On the basis of the spread of every dominant COVID-19 variant, we considered four temporal waves of disease.

The first wave (from April 2020 to June 2020) included 58 patients (male: 30; female: 28; mean age: 69 years) who tested positive for the wild-type lineage.

The second wave (from September 2020 to December 2020) incorporated 167 patients (male: 104, female: 63; mean age: 65 years) and the dominant strain was the alpha one, also called the “English variant”.

During the third wave (from January 2021 to April 2021) the circulating SARS-CoV-2 viral strain was characterized by the transition from the English variant to the delta variant, also called the ‘’Indian variant’’, with this latter being dominant during the fourth (current) wave (from June 2021 to October).

Cohorts of 119 (male: 82, female: 37; mean age: 63 years) and 123 patients (male: 82, female: 37; mean age: 63 years), respectively, were included (Table 1 and Table 2).

For each wave, we analysed the first chest CT scan performed upon admission at the “Policlinico di Bari” COVID Hospital, Italy, from March 2020 to October 2021.

We focused on typical COVID-19 patterns such as GGOs, consolidation and subpleural bands, and we also considered one unusual pattern, namely pleural effusion.

Bilaterality of pulmonary disease was found in 100% of the evaluated patients.

**Figure 2:**
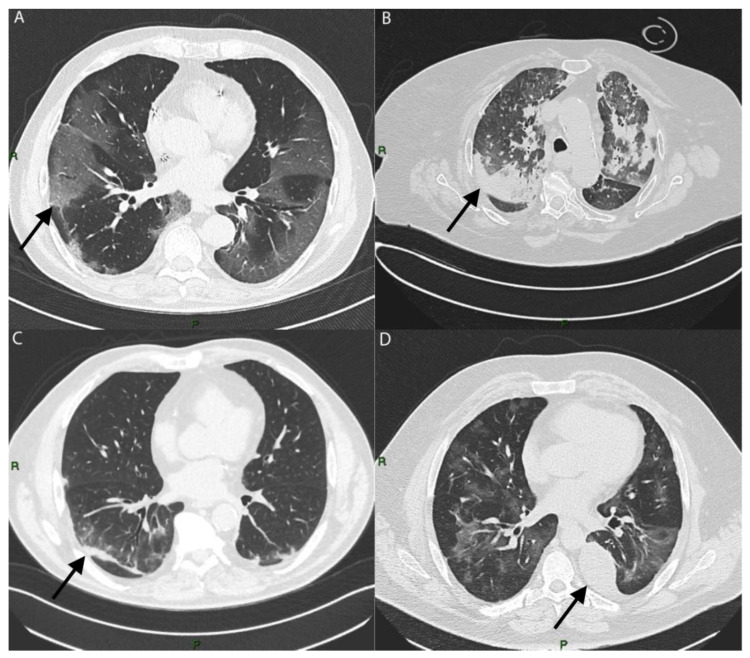
Axial images of different evaluated patterns of COVID-19 (each marked with arrow keys): (**A**) bilateral ground glass (GGOs); (**B**) consolidation; (**C**) subpleural bands; (**D**) pleural effusion.

The typical patterns observed were: GGOs (defined as hazy increased opacities of the lung with the preservation of bronchovascular margins), consolidation (a homogeneous increase in pulmonary parenchymal attenuation that obscures the margins of vessels and airway walls), GGOs plus consolidations (consolidations superimposed on a background of GGOs), GGOs plus subpleural bands (subpleural bands defined as thin curvilinear opacities with 1–3 mm thickness, lying less than 1 cm from and parallel to the pleural surface) and parenchymal bands (defined as linear opacities, usually 1–3 mm thick and up to 5 cm long that usually extend to the visceral pleura) [19] (Figure 2).

In each wave, the predominant patterns were GGOs (first wave: 89.6%; second wave: 91.6%; third wave: 100%; fourth wave: 83%).

The occurrence rates of consolidation and subpleural bands were similar between all waves.

**Figure 3:**
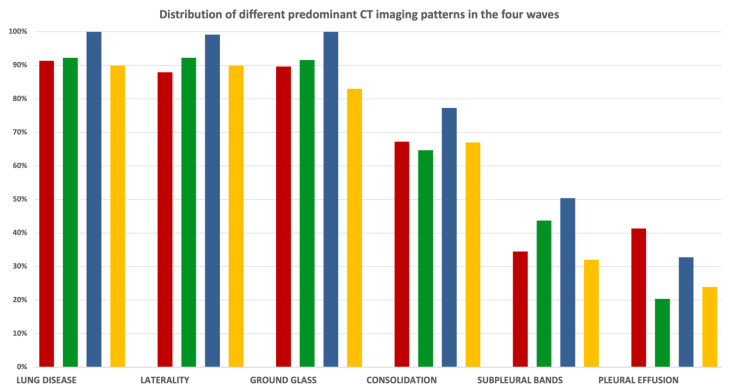
Percentage distribution of predominant CT patterns in the four waves.

Pleural effusion, which is an unusual manifestation of COVID-19 disease, was more present in the very early stages of the disease, in particular during the first wave (first wave: 41.4%; second wave: 20.4%; third wave: 32.8%; fourth wave: 23.9%) (Figure 2D; percentages reported in Figure 3, Figure S1 and Table S2).

**Figure 4:**
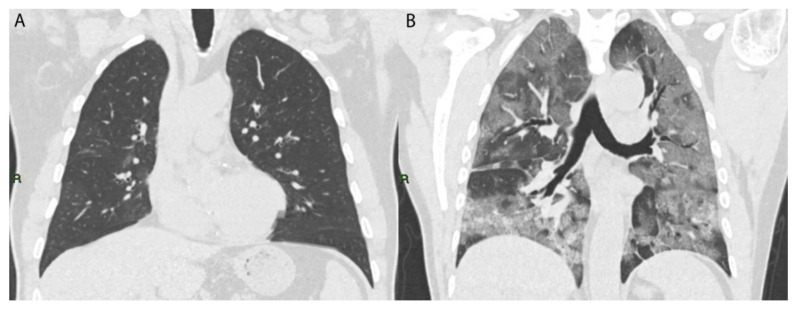
Coronal TC image of two COVID-19 patients: (**A**) patient with low CT score (5/25); (**B**) patient with high CT score (24/25).

To estimate the involvement of lung lesions, we used a semi-quantitative CT-score system that ranged from 0 to 5 points for each lobe (Figure 4).

**Figure 5:**
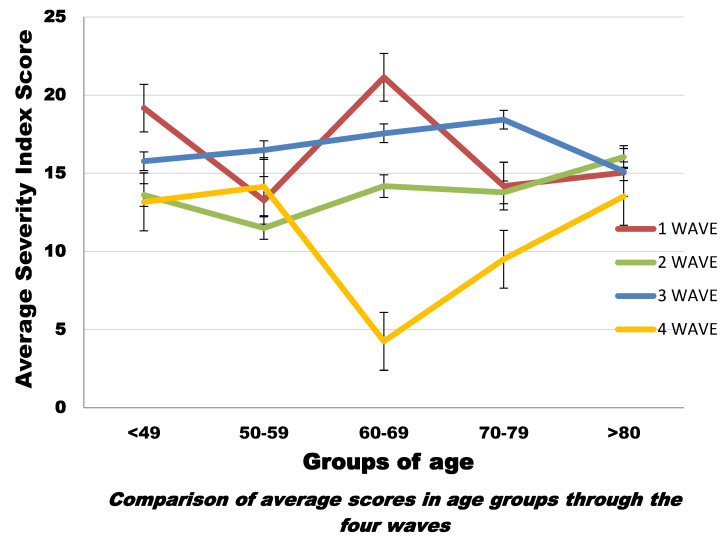
Graphic representation of mean CT scores in age groups through all waves of the disease.

**Figure 6:**
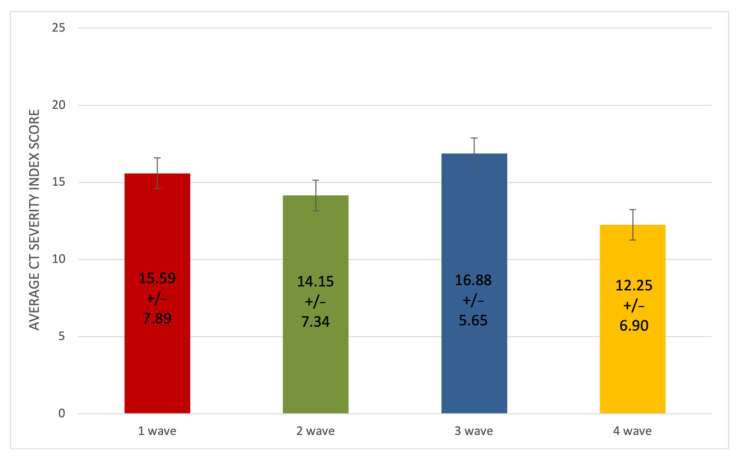
Comparison of CT scores (and ±SD) through the four waves of the disease.

We also compared the mean CT scores for each age group between waves (Figure 5 and Table S1). For most age groups, the average CT severity scores across the four waves were within the same order of magnitude. There was also no major difference among the mean CT scores (first wave: 15.59; second wave: 14.15; third wave: 16.88; fourth wave: 12.24) (*p* < 0.001) (Figure 6).

In addition, the mean age decreased over time and males were more affected than females and developed the disease earlier (first wave: mean age in males—62; mean age in females—76; second wave: mean age in males—62; mean age in females—69; third wave: mean age in males—61; mean age in females—66; fourth wave: mean age in males—63; mean age in females—66 (*p* < 0.002)).

## 4. Discussion

This study analysed the chest CT patterns of 461 patients with COVID-19 from March 2020 until October 2021. We divided the time course into four stages according to the dominant variant in each one.

Following the current literature [21], we considered COVID-19 CT manifestations as typical and atypical (such as pleural effusion); moreover, we chose to evaluate the pulmonary involvement (expressed by the semiquantitative CT score) and the extent of disease to both lungs. Similarly to current reports [21], the bilateral lung involvement was depicted.

In particular, bilateral subpleural GGOs, found mainly in the lower lobes, was the most frequent CT finding at admission. Their prevalence also reached 100% of cases during the third wave (Figure 3, Figure S1 and Table S2), but appeared not to be linked to the hypothetical viral genotype of the investigated wave.

During the early stages of the disease, due to the poor knowledge about the emerging COVID-19 pneumonia, people mostly showed up to the emergency room with severe symptoms, and this was corroborated by the CT score mode being higher for these stages than for the late stages of the pandemic (first wave average CT mode: 25; third wave average CT mode: 20), which also described a worse CT scenario.

Another interesting finding was given by pleural effusion, which is an unusual manifestation of COVID-19 disease [21] and an initial sign of inflammation; this was most recurrent in the very early stages of the disease, in particular during the first wave, suggesting that patients showed up more to the emergency room with an acute inflammatory phase of disease (percentage of pleural effusion: first wave—41%; fourth wave—24%) probably because they did not have access to home care due to the novelty of the disease (Figure 6). Pleural effusion decreased during the remaining waves, perhaps due to the improvement of home care management. This led patients to present to the ER later, only when they could no longer treat the disease at home.

Predominant CT patterns did not show any significant differences with variations in age (Figure 5 and Table S1).

The main COVID-19 CT patterns did not show any remarkable differences among the different waves, so our experiences underline that CT imaging is not useful to discriminate between different CoV-2 genotypes (Figure 3, Figure S1 and Table S2).

It is relevant to point out that during the fourth wave, the CT findings appeared to be slightly less severe as compared to the other waves. This could probably be explained by an increase in the number of patients immunized/vaccinated against COVID-19 and by improved management of home-based medical care for symptoms.

It is worth mentioning the limitations of this study: we evaluated only non-contrast CT scans at admission, so other findings such as pulmonary embolism were not evaluated. There were selection biases—it is unknown whether people had prior pulmonary disease, especially a history of smoking, and this could have led to an overestimation of the impact of COVID-19 in the CT examinations.

Not all virus sequencing from enrolled patients is disposable. Hence, we based our assumptions on the predominant statistical variant for every wave.

This aspect emerged above all during the third wave, where we saw a transition between two different variants, which was challenging to classify.

The study was also based on the chest CT results of the first patients admitted to the ER.

It was difficult to set up a timeline from the onset of symptoms to the first CT scan because of differences in timing arising from the varying severity of patients’ conditions.

In addition, vaccination rates are increasing nowadays thanks to the spreading of world vaccination campaigns and this could have interfered with COVID-19 disease history, transmission and manifestation.

To conclude, the first CT main pattern (GGOs) was the same in every wave, proving that CT patterns are not pathognomonic in terms of predicting which virus genotype started the illness. However, CT played an important role in diagnosis, in the evaluation of disease severity and in the guiding therapy, regardless of COVID-19 genotype. It was, in fact, a valuable tool to quantify the lung involvement and the pulmonary alteration features, even if there were no statistical differences during the four waves examined, showing that pulmonary damage and CT patterns were not linked to the virus genome.

## Figures and Tables

**Table 1:** Population sex ratio.

PATIENTS	FEMALE (*n*)	FEMALE (%)	MALE (*n*)	MALE (%)
**FIRST WAVE**	28	15.9	30	10.5
**SECOND WAVE**	63	35.8	104	36.5
**THIRD WAVE**	37	21.0	82	28.8
**FOURTH WAVE**	48	27.3	69	24.2

**Table 2:** Population demographics: population mean age and standard deviation (SD).

PATIENTS	MEAN AGE (±SD)	MALE MEAN AGE (±SD)	FEMALE MEAN AGE (±SD)
**FIRST WAVE**	68.8 ± 17.5	62 ± 15.1	76 ± 17.4
**SECOND WAVE**	69.7 ± 15.0	67.4 ± 14.9	73.7 ± 14.7
**THIRD WAVE**	63 ± 14.6	61.3 ± 13.8	66.8 ± 16.1
**FOURTH WAVE**	63.8 ± 19.5	62.7 ± 21.2	65.9 ± 20.7

## Data Availability

Interdisciplinary Department of Medicine, Section of Radiology and Radiation Oncology, University of Bari “Aldo Moro”, Bari, Italy.

## Supplementary Materials

The following supporting information can be downloaded at: https://www.mdpi.com/article/10.3390/microorganisms10040796/s1, Table S1: Graphic representation of mean CT-score trend in age groups through the four waves of disease. Figure S1: Percentage distribution of predominant CT patterns in the four waves. Table S2: Percentage distribution of predominant CT patterns in the four waves.
