# Supplementary material for: COVID-19 in Italy: Comparison of CT Findings from Time Zero to the Delta Variant
Source: Microorganisms. 2022 Apr 9;10(4):796. doi: 10.3390/microorganisms10040796 (PMC9026540; doi:10.3390/microorganisms10040796)
Supplement: Supplementary file 1 [file microorganisms-10-00796-s001.zip › microorganisms-1605499-supplementary.pdf]

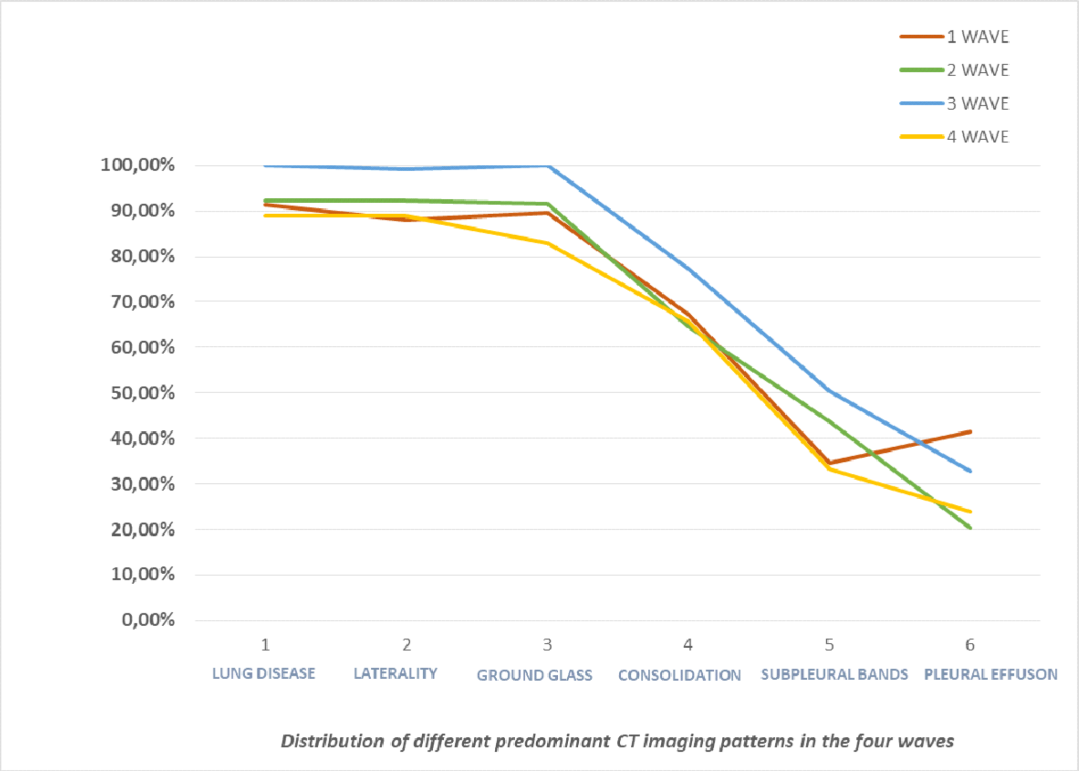

Figure S1. Percentage distribution of predominant CT patterns in the four waves.

Table S1. Graphic representation of mean CT-scores in age groups through all waves of disease.

GROUPS OF AGE	FIRST WAVE	SECOND WAVE	THIRD WAVE	FOURTH WAVE
**<49**	19.17	13.61	15.77	13.17
**50-59**	13.27	11.50	16.48	14.14
**60-69**	21.14	14.18	17.56	4.25
**70-79**	14.18	13.78	18.43	9.50
**>80**	15.05	16.04	15.13	13.53

Table S2. Percentage distribution of predominant CT patterns in the four waves.

CT FINDINGS	FIRST WAVE	SECOND WAVE	THIRD WAVE	FOURTH WAVE
**LUNG DISEASE**	91.38	92.22	100	88.89
**LATERALITY**	87.93	92.22	99.16	88.89
**GROUND GLASS**	89.66	91.62	100	83
**CONSOLIDATION**	67.24	64.67	77.31	65.80
**SUBPLEURAL BANDS**	34.48	43.71	50.42	33.30
**PLEURAL EFFUSION**	41.38	20.36	32.77	23.90
